# Supplementary material for: Identification of Elite Alleles and Candidate Genes for the Cotton Boll Opening Rate via a Genome-Wide Association Study
Source: Int J Mol Sci. 2025 Mar 17;26(6):2697. doi: 10.3390/ijms26062697 (PMC11943326; doi:10.3390/ijms26062697)
Supplement: Supplementary file 1 [file ijms-26-02697-s001.zip › Figures S1-S3.pdf]

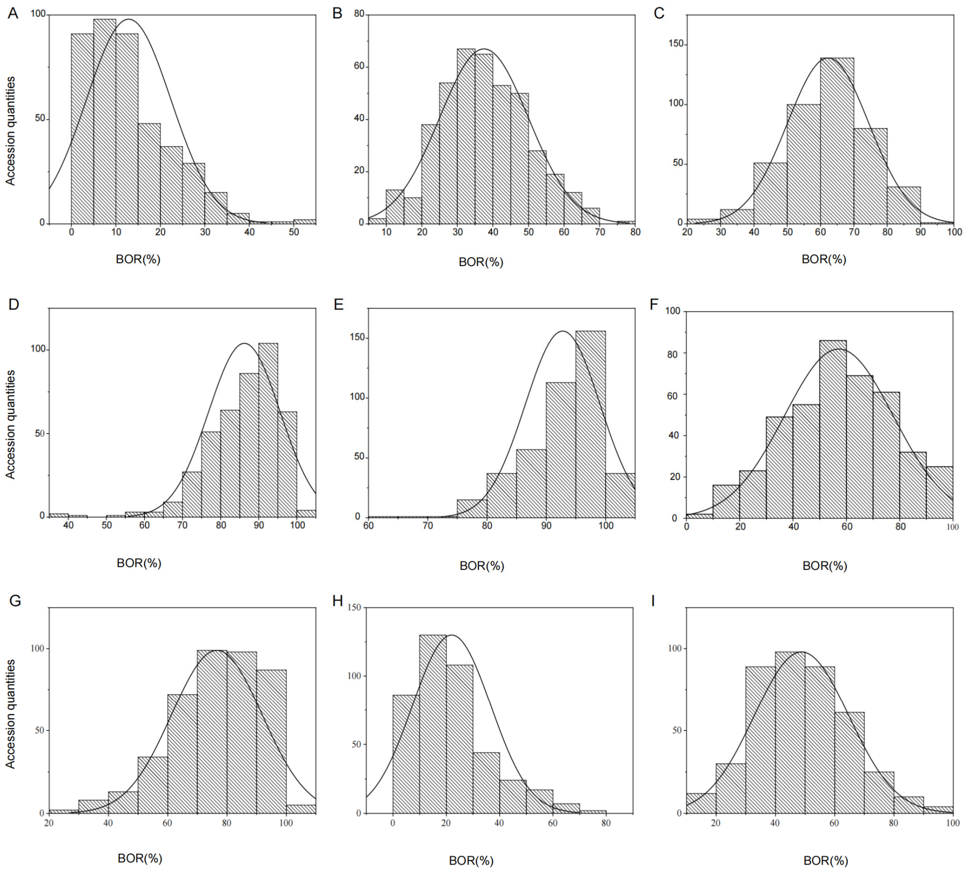

Figure S1. Frequency histogram of the BOR of 418 upland cotton accessions in 2020. A, B and C, SHZ-20 (8.30), SHZ-20 (9.6) and SHZ-20 (9.14). D, E and F, SHZ-20 (9.20), SHZ-20 (9.24) and KEL-20 (9.24). G, H and I, KEL-20 (9.30), DH-20 (9.8) and DH-20 (9.21).

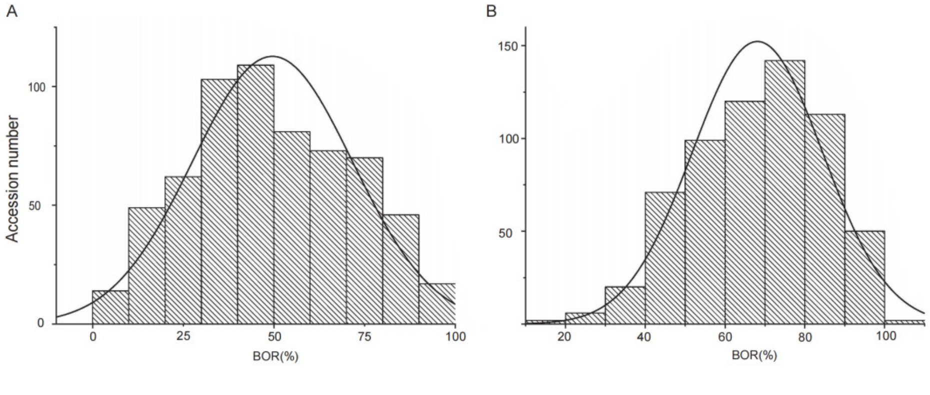

Figure S2. Frequency histogram of the BOR of 418 upland cotton accessions in 2021. A and B, SHZ-21 (9.14) and KEL-21 (9.24).

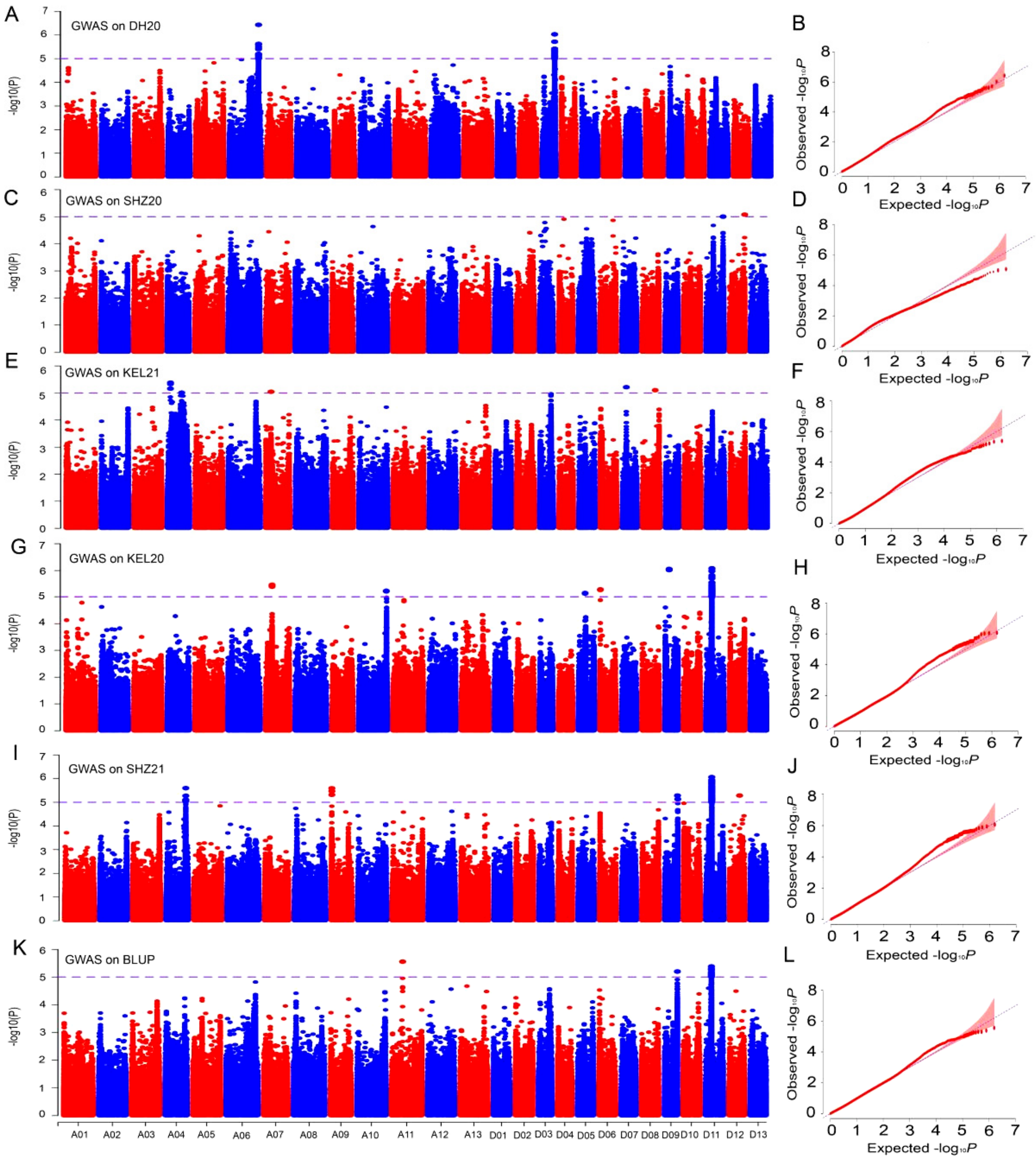

Figure S3. Significant Manhattan and quantile–quantile (Q–Q) plots of the GWAS results. A and B, Manhattan and Q–Q plots for DH-20. C and D, Manhattan and Q–Q plots for SHZ-20. E and F, Manhattan and Q–Q plots for KEL-21. G and H, Manhattan and Q–Q plots for KEL-20. I and J, Manhattan and Q–Q plots for SHZ-21. K and L, Manhattan and Q–Q plots for BLUP.
